# Factors influencing the functional status of aortic valve in ovine models supported by continuous‐flow left ventricular assist device

**DOI:** 10.1111/aor.14207

**Published:** 2022-03-03

**Authors:** Xin‐Yi Yu, Jian‐Wei Shi, Yi‐Rui Zang, Jie‐Min Zhang, Zhi‐Gang Liu

**Affiliations:** ^1^ Department of Cardiovascular Surgery, TEDA International Cardiovascular Hospital Chinese Academy of Medical Sciences & Graduate School of Peking Union Medical College Tianjin China; ^2^ Laboratory Animal Center, TEDA International Cardiovascular Hospital Chinese Academy of Medical Sciences & Graduate School of Peking Union Medical College Tianjin China

**Keywords:** aortic valve, influencing factors, LVAD, nomogram, predictive model

## Abstract

**Objectives:**

An acute animal experiment was performed to observe factors influencing the functional status of the aortic valve functional status after continuous‐flow left ventricular assist device (CF‐LVAD) implantation in an ovine model, and a physiologic predictive model was established.

**Methods:**

A CF‐LVAD model was established in Small Tail Han sheep. The initial heart rate (HR) was set to 60 beats/min, and grouping was performed at an interval of 20 beats/min. In all groups, the pump speed was started from 2000 rpm and was gradually increased by 50–100 rpm. A multi‐channel physiological recorder recorded the HR, aortic pressure, central venous pressure, and left ventricular systolic pressure (LVSP). A double‐channel ultrasonic flowmeter was used to obtain real‐time artificial vascular blood flow (ABF). A color Doppler ultrasound device was applied to assess the aortic valve functional status. Multivariate dichotomous logistic regression was used to screen significant variables for predicting the functional status of the aortic valve.

**Results:**

Observational studies showed that ABF and the risk of aortic valve closure (AVC) were positively correlated with pump speed at the same HR. Meanwhile, the mean arterial pressure (MAP) was unaltered or slightly increased with increased pump speed. When the pump speed was constant, an increase in HR was associated with a decrease in the size of the aortic valve opening. This phenomenon was accompanied by an initial transient increase in the ABF and MAP, which subsequently decreased. Statistical analysis showed that the AVC was associated with increased pump speed (OR = 1.02, 95% CI = 1.01–1.04, *p* = 0.001), decreased LVSP (OR = 0.95, 95% CI = 0.91–0.98, *p* = 0.003), and decreased pulse pressure (OR = 0.82, 95% CI = 0.68–0.96, *p* = 0.026). ABF or MAP was negatively associated with the risk of AVC (OR < 1). The prediction model of AVC after CF‐LVAD implantation exhibited good differentiation (AUC = 0.973, 95% CI = 0.978–0.995) and calibration performance (Hosmer–Lemeshow χ^2^ = 9.834, *p* = 0.277 > 0.05).

**Conclusions:**

The pump speed, LVSP, ABF, MAP, and pulse pressure are significant predictors of the risk of AVC. Predictive models built from these predictors yielded good performance in differentiating aortic valve opening and closure after CF‐LVAD implantation.

## Graphical abstract

HeartCon Magnetic and Hydrodynamic Levitation Blood Pump (RocketHeart, Tianjin, China) was used in the construction of CF‐LVAD assisted animal models. With CF‐LVAD support, the risk of aortic valve closure can be predicted by the combined application of pump speed, left ventricular systolic pressure, artificial vascular blood flow, mean arterial pressure and pulse pressure. The model exhibited good differentiation.

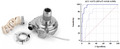

## 1 INTRODUCTION

Continuous‐flow left ventricular assist device (CF‐LVAD) has become the mainstay for treatment of end‐stage heart failure serving as a bridge to heart transplantation and destination therapy.^1^, ^2^ Nevertheless, there is a risk of shortened opening time or even complete closure of the aortic valve, as the device provides flow directly from the left ventricle to the aorta, which leads to increased cardiac output, decreased left ventricular pressure while increasing the aortic valve pressure load.^3^, ^4^ An increasing body of evidence suggests that aortic valve closure (AVC) is associated with multiple postoperative complications, such as aortic valve fusion and insufficiency, leaflet thickening, and thrombus formation.^3^, ^5^ Besides, the resultant AVC may be persistent, which can affect the pulsatile flow generated from the natural ventricular contraction. Merwe et al. hypothesized that the weakening or disappearance of the pulsatile flow is responsible for early gastrointestinal complications secondary to CF‐LVAD implantation.^6^ In this regard, it should be borne in mind that long‐term continuous blood flow is not conducive to healthy splanchnic circulation and increases the risk of bleeding. Consistently, it has been documented that lower pulsatility is associated with a higher risk of bleeding.^7^, ^8^

Moreover, the complications secondary to aortic valve dysfunction can affect the mortality, rehospitalization rate, and hospital stay of patients supported with CF‐LVAD.^5^, ^6^ Prior studies on CF‐LVAD and the functional status of the aortic valve, predominantly conducted univariate analyses, epidemiologic analyses of related complications, discussed the management, and treatment strategies and conducted simulations of in vitro circulation.^1^, ^5^, ^9^, ^10^ Accordingly, it remains unclear whether there is a direct relationship between aortic valve functional status and pump speed, artificial vascular blood flow (ABF) as well as various physiologic indices, such as heart rate (HR), mean arterial pressure (MAP), pulse pressure (PP), left ventricular pressure (LVP), and central venous pressure (CVP).

In the present study, the CF‐LVAD model was established in Small Tail Han sheep. Acute animal experiments were performed to observe the functional status of the aortic valve after CF‐LVAD implantation. Additionally, multivariate analysis was conducted to discuss the significant factors that influenced aortic valve functional status. Finally, we established a physiologic predictive model.

## 2 MATERIALS AND METHODS

### 2.1 Experimental device

**FIGURE 1:**
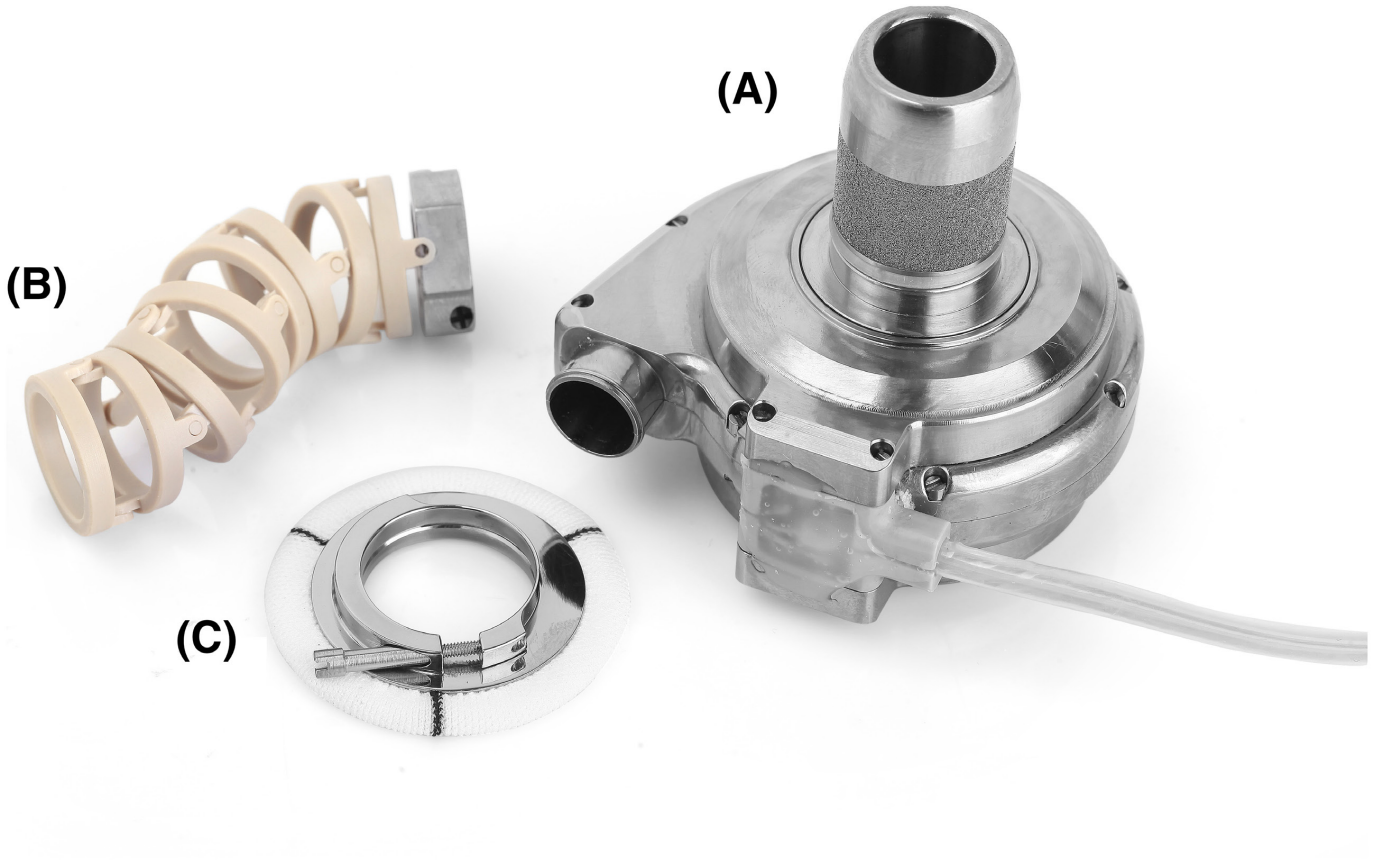
HeartCon implantable magnetic and hydrodynamic levitation blood pump and attachments. (A) blood pump; (B) artificial vascular protective stent; (C) sewing ring

**FIGURE 2:**
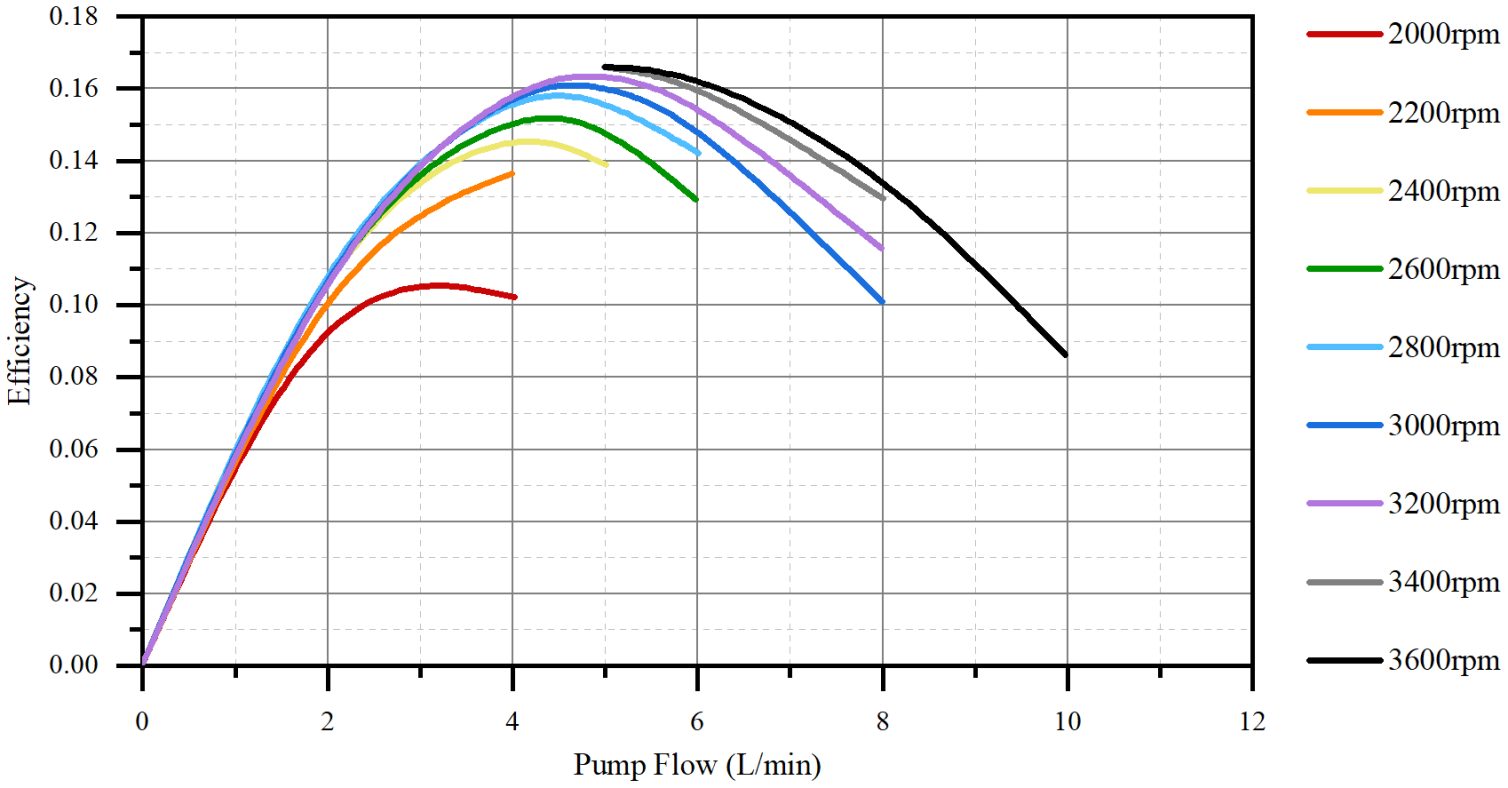
The efficiency curve of HeartCon

**FIGURE 3:**
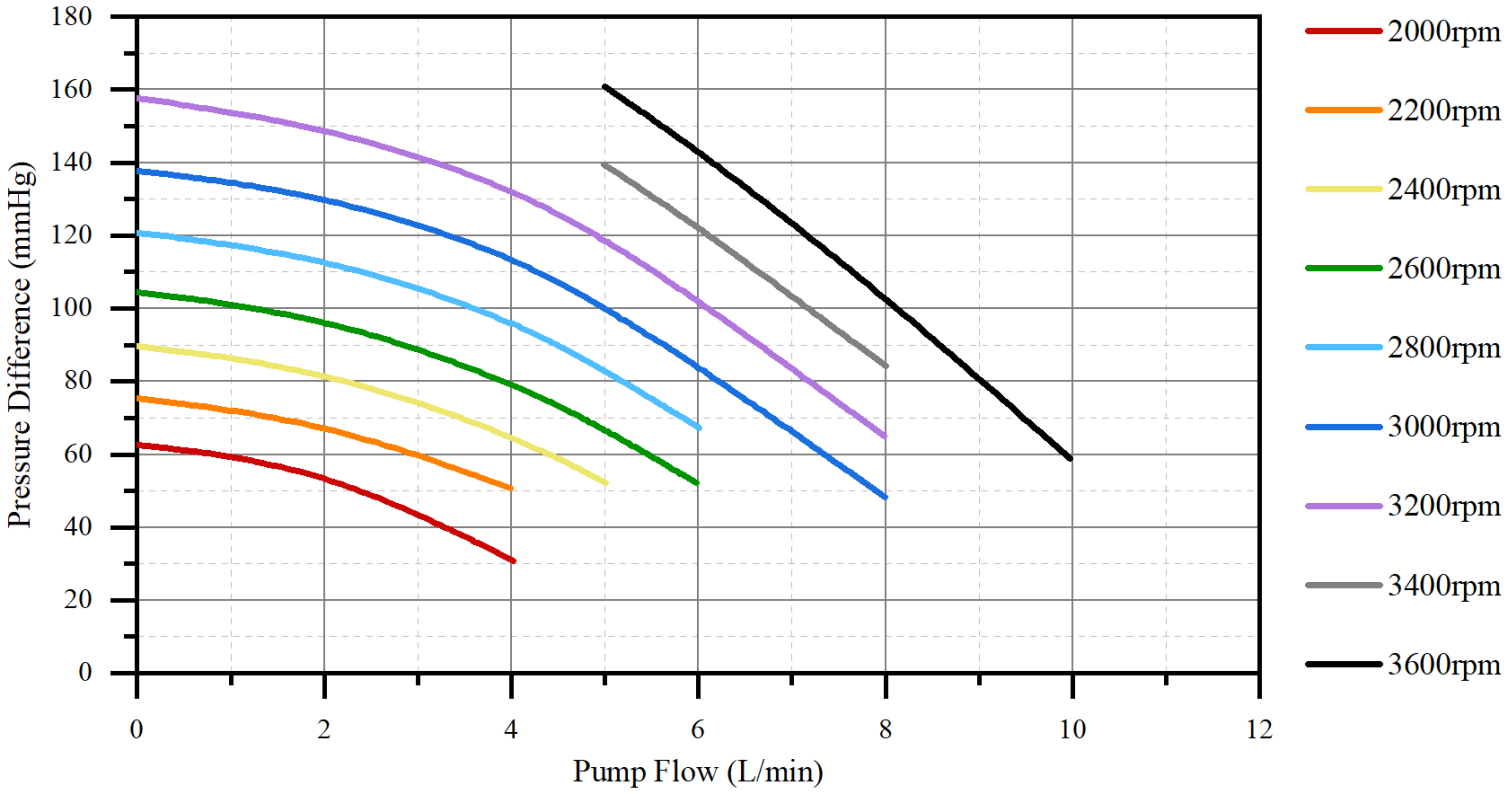
The flux characteristic curve of HeartCon. Pressure difference = Outlet pressure − Inlet pressure

HeartCon Magnetic and Hydrodynamic Levitation Blood Pump (RocketHeart, Tianjin, China) with good biocompatibility, consisting of a body made of titanium alloy weighing 190 g, a radius of gyration of 18 mm, and a maximum auxiliary flow of 10 L/min, was used (Figures 1A, 2, and 3).^11^ Attachments included an artificial vascular protective stent (Figure 1B), sewing ring (Figure 1C), ventricular punch, percutaneous lead device, etc. The background monitoring system was operated to obtain real‐time feedback of the animal's HR and the device's voltage, current, and auxiliary flow.

### 2.2 Animal model

Five healthy adult male Small Tail Han sheep weighing between 54 and 62 kg were used in the study. All the five sheep were housed in separate cages for 2 weeks, and no abnormal clinical signs or blood indicators were observed. The animal experiment was conducted at the Laboratory Animal Center of TEDA International Cardiovascular Hospital and was approved by our Institutional Animal Care and Use Committee. All animals received humane care strictly following the Regulations for the Administration of Affairs Concerning Experimental Animals (2017.03.01 edition) published by the State Council of the People's Republic of China.

### 2.3 Anesthesia and surgical preparation

The five sheep were subjected to 24‐h fasting and 20‐h water deprivation prior to operation. After animal sedation by intramuscular injection of xylazine (1.0 mg/kg), the surgical site was exposed, and the neck skin was prepared. Vascular access was obtained through the great saphenous vein, and an intravenous bolus of propofol (60–200 mg) was injected. Tracheal intubation was then performed with parameters including tidal volume 8–12 ml/kg/min, frequency 12–20 times/min, inspired oxygen concentration 40%–100%, positive end‐expiratory pressure (PEEP) 5–10 mm Hg (1 mm Hg = 0.133 kPa) and sevoflurane inhalation 2%–4%. A nasogastric tube was inserted for decompression. The arterial puncture was achieved through the left ear to obtain arterial access, and central venous access was obtained via puncture of the left internal jugular vein. Anesthesia was maintained by continuous infusion of dexmedetomidine (0.5–1 mg/kg/h) and succinylcholine (50–100 mg/h).

### 2.4 Operative procedures

A thoracotomy was performed at the left fifth intercostal space, and a lidocaine drip (2 mg/min) was initiated to prevent arrhythmias. The pericardium was incised from the apex to the pulmonary artery, and the heart was suspended in a pericardial cradle. The site for pump placement was determined according to the apex position. After obtaining a whole‐blood clotting time greater than 450 s by intravenous bolus injection of 1.0 mg/kg heparin, the descending thoracic aorta was dissected for outflow graft anastomosis. A partial occlusion clamp was applied, and the pump's 10‐mm outflow graft was sewn end‐to‐side to the descending aorta with 4‐0 Prolene sutures. Hemostasis of the anastomotic site was obtained to reduce the effect of excessive bleeding on capacity loading. The sewing ring was attached to the left ventricular apex with eight interrupted sutures. The left pleural cavity was filled with warm normal saline. A cruciform incision was made on the beating heart. Then a ventricular punch was advanced through the ventricular sewing ring, the left ventricular core was removed, and the pump was inserted into the left ventricular cavity and secured. The pump was started at 2000 rpm; then, the artificial blood vessel was opened after full gas exhausting.

### 2.5 Research methods

Following CF‐LVAD placement, HR control was achieved via drugs (such as isoproterenol) or a temporary cardiac pacemaker. The initial HR was set to 60 beats/min, and grouping was performed at an interval of 20 beats/min. The pump was started at 2000 rpm in all groups, which gradually increased by 50–100 rpm. During the experiment, no intervention was required if the animal presented with stable vital signs and blood circulation. Moreover, in case of capacity loading loss, succinylated gelatin was supplemented by internal jugular vein injection (such as urinary volume increase). The experiment was terminated when AVC was observed. Data collection was performed 10 min after each pump speed adjustment (when the animal presented with stable vital and circulatory signs). Consequently, the result was ensured to be independent to avoid mutual influences between adjacent settings.

A multi‐channel physiological recorder (MP150 Biopac, USA) was used to record the HR, aortic pressure (AOP) (the measurement point was aortic root), CVP, and LVP. A double‐channel ultrasonic flowmeter (Transonic, USA) was operated to obtain real‐time ABF (the measurement point was pump outflow graft). A color doppler ultrasound (Philips5500, Netherlands) was used to obtain aortic valve functional status from the heart surface. Aortic valve opening (AVO) was defined as complete opening and intermittent opening (at least one opening observed during three contractions), while AVC was defined by persistent complete closure during three contractions.

### 2.6 Statistical analysis

SPSS (ver 26.0, USA) and R software (ver 4.0.5, USA) were used to perform data analysis. Non‐parametric quantitative data were expressed as the medium or interquartile range (IQR) and compared by Wilcoxon rank‐sum test. Normally distributed quantitative data were expressed as mean ± standard deviation and compared by the two‐sample independent t‐test. Durbin‐Watson test was conducted to identify the independence of each variable. Multivariate dichotomous logistic regression was used to screen variables with significant value in predicting aortic valve functional status, according to the stepwise forward regression based on the maximum‐likelihood estimation method. A *p*‐value < 0.05 was statistically significant.

## 3 RESULTS

All five sheep exhibited stable vital signs after anesthesia, with HR 96.75 ± 37.27 beats/min, MAP 88.08 ± 17.50 mm Hg, PP 18.25 ± 1.7 mm Hg, and CVP 4.75 ± 3.77 mm Hg. The arterial blood gas analysis revealed that the pH was 7.57 ± 0.04, PCO_2_ 33.00 ± 11.14 mm Hg, PO_2_ 345.74 ± 124.88 mm Hg, BE 7.10 ± 7.15, HCO_3_^−^ 29.3 ± 8.86 mmol/L, and tHb 11.78 ± 1.47 g/dL. During the cardiac ultrasound, there were no calcifications or malformations of the aortic valve, while healthy valve leaflets operated at a 1:1 open/closure ratio. The left ventricular end‐diastolic volume was 39.50 ± 8.96 mL and the left ventricular ejection fraction was 66.25 ± 9.91. A total of 97 observational data were observed during the experiment. A Durbin‐Watson test value of 1.991 (close to 2) implied the independence of each variable. Moreover, changes in pump speed led to small changes in vital signs in groups with different HR. The capacity and fluid infusion volume were determined comprehensively by combining the urinary volume, intraoperative blood loss, and CVP.

**FIGURE 4:**
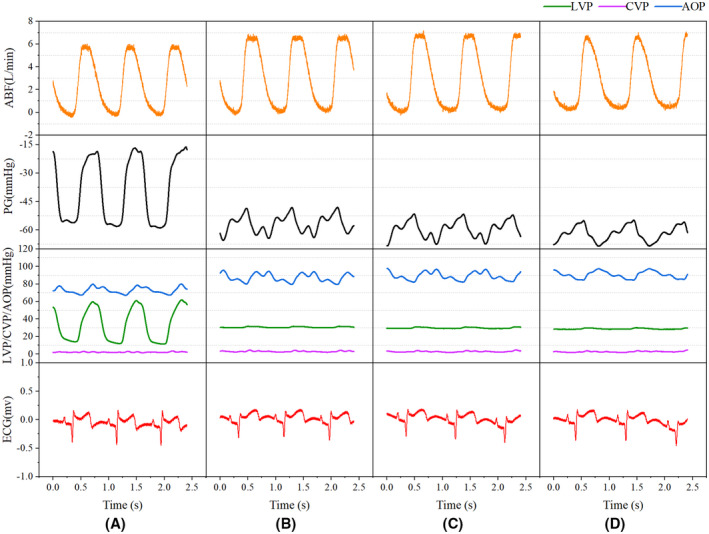
The functional status of the aortic valve with changing pump speed at a constant HR of 70 beats/min. (A) 2100 rpm pump speed, AVO; (B) 2300 rpm pump speed, AVO; (C) 2350 rpm pump speed, AVO; (D) 2400 rpm pump speed, AVC. ABF, artificial vascular blood flow; AOP, aortic pressure; AVC, aortic valve closure; AVO, aortic valve opening; HR, heart rate; PD, pressure difference

**FIGURE 5:**
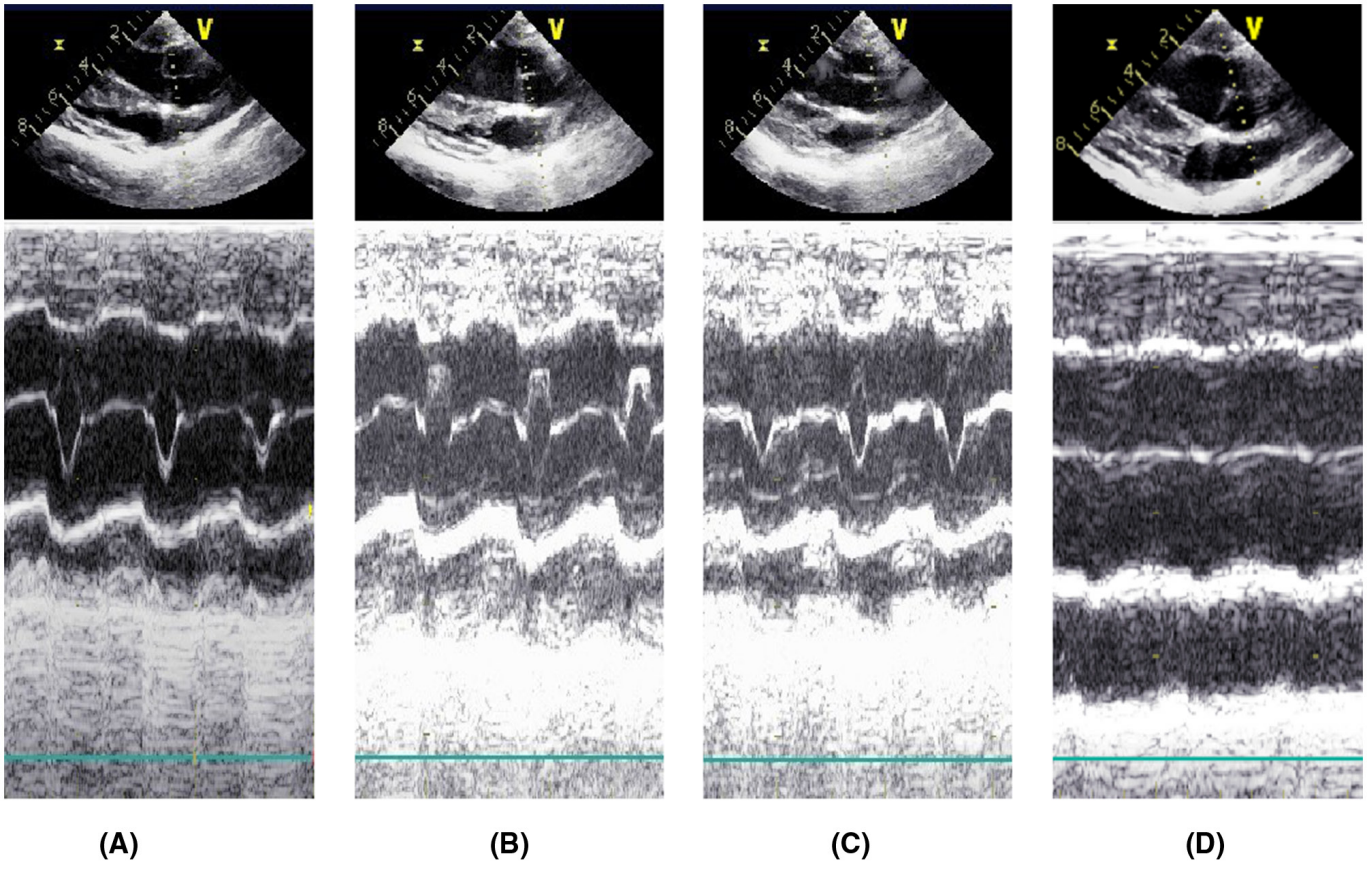
M‐mode echocardiography of the functional status of the aortic valve at a constant HR of 70 beats/min. All ultrasound examinations showed no signs of aortic regurgitation. Aortic valve leaflets open/close 1:1 in A–C. complete closure of the aortic valve leaflets in D. (A) 2100 rpm pump speed, AVO; (B) 2300 rpm pump speed, AVO; (C) 2350 rpm pump speed, AVO; (D) 2400 rpm pump speed, AVC

**FIGURE 6:**
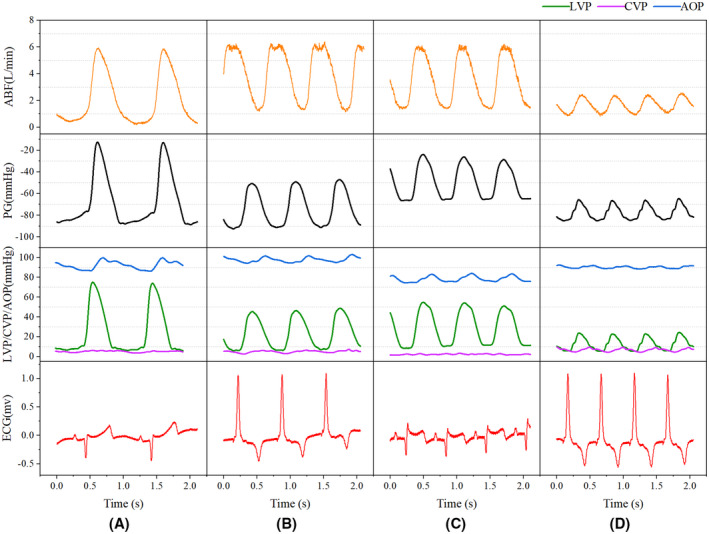
The functional status of aortic valve with changing HR at a constant pump speed of 2400 rpm. (A) 61 beats/min HR, AVO; (B) 90 beats/min HR, AVO; (C) 104 beats/min HR, AVO; (D) 120 beats/min HR, AVC. ABF, artificial vascular blood flow; AOP, aortic pressure; AVC, aortic valve closure; AVO, aortic valve opening; HR, heart rate; PD, pressure difference

At the same HR, the ABF, and the risk of AVC were positively correlated with pump speed, while the MAP was unchanged or slightly increased with an increase in pump speed. Under these circumstances, the pressure difference (PD) between LVP and MAP decreased with AVC (Figures 4 and 5). When the pump speed was constant, an increase in HR could decrease the size of the aortic valve opening. Meanwhile, the ABF, MAP, and PD exhibited a transient increase followed by a decrease (Figure 6). Regarding CVP, regular waveforms were observed in each group, irrespective of the pump speed and HR. Moreover, we found that the pump speed, HR and ABF associated with AVC were not the same (Figures 4 and 6), which could be attributed to the heterogeneity among the sheep observed.

All observational data were grouped based on the opening and closure of the aortic valve. Then, the data of the two groups were compared (Table 1). Although the HR was higher in the AVC group compared to the AVO group (*p* < 0.05), the PD, PP, MAP, left ventricular systolic pressure (LVSP), and ABF were lower (*p*_*s*_ < 0.05).

**TABLE 1:** Between‐group comparison

Variables	AVO (*n* = 51)	AVC (*n* = 46)	*t*/*Z*	*p*
PRS [rpm, median (IQR)]	2400.00 (2325.00, 2400.00)	2400.00 (2400.00, 2450.00)	853.50	0.013
HR [beats/min, median (IQR)]	90.00 (80.00, 124.50)	120.00 (85.00, 235.00)	844.50	0.018
CVP [mm Hg, median (IQR)]	7.00 (5.50, 7.00)	7.00 (5.00, 9.00)	892.50	0.040
PP [mm Hg, median (IQR)]	11.00 (7.00, 16.00)	6.00 (3.25, 10.00)	1697.00	<0.001
MAP [mm Hg, median (IQR)]	85.00 (80.83, 92.00)	77.67 (62.08, 89.92)	1538.50	0.008
LVSP [mm Hg, median (IQR)]	95.00 (83.00, 104.00)	42.50 (24.00, 57.00)	2001.00	<0.001
PD [mm Hg, median (IQR)]	9.67 (−2.33, 17.17)	−32.00 (−53.42, −8.33)	464.00	<0.001
ABF (L/min, mean ± SD)	3.20 ± 0.70	2.36 ± 0.95	4.89	<0.001

Abbreviations: ABF, artificial vascular blood flow; AVC, aortic valve closure; AVO, aortic valve opening; CVP, central venous pressure; HR, heart rate; IQR, interquartile range; LVSP, left ventricular systolic pressure; MAP, mean arterial pressure; PD, pressure difference; PP, pulse pressure; PRS, pump rotate speed.

**FIGURE 7:**
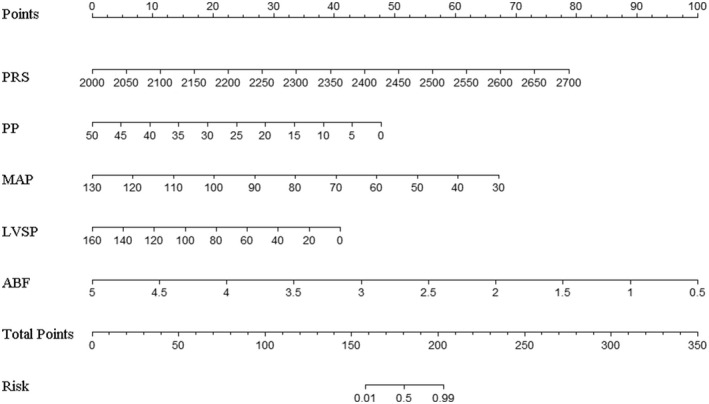
Nomogram. ABF, artificial vascular blood flow; LVSP, left ventricular systolic pressure; MAP, mean arterial pressure; PP, pulse pressure; PRS, pump rotate speed

**FIGURE 8:**
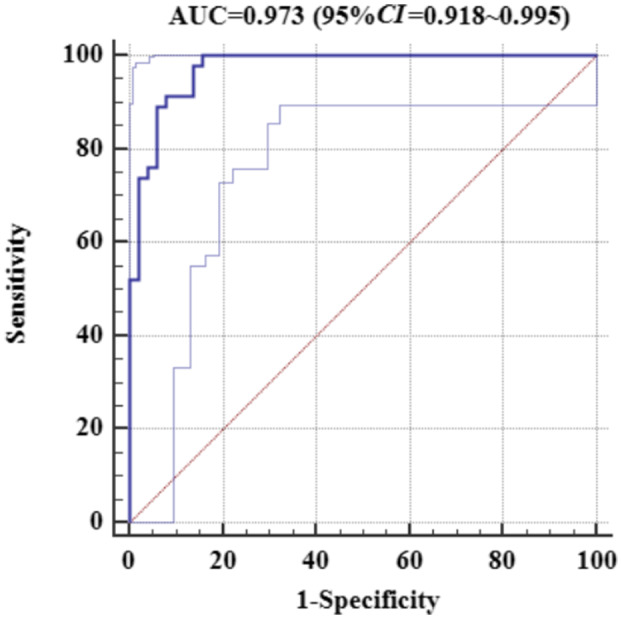
ROC curve analysis

Dichotomous logistic regression analysis showed that PD was a predictor of aortic valve functional status (OR = 0.97, 95% CI of OR = 0.95–0.98, *p* < 0.05). The accuracy (consistency) of PD in predicting the risk of AVC was 75.3%, with a specificity of 84.3% and a sensitivity of 65.2%. ROC curve analysis yielded an area under the curve (AUC) value of 0.802 (95% CI = 0.709–0.876). Multivariate dichotomous logistic regression showed that pump speed, ABF, LVSP, MAP, and PP had statistical value in predicting aortic valve functional status using forward stepwise regression based on the maximum‐likelihood estimation method (*p* < 0.05, Table 2). Interestingly, we found that the pump speed was positively correlated with the risk of AVC, while LVSP, PP, ABF, and MAP were negatively correlated. A logistic regression model (Figure 7) was built as shown below:

$$\begin{array}{c} \text{Logit}\left(P\right)=-24.043+0.023\times \mathrm{PRS}-0.194\times \mathrm{PP}-0.137\\ \;\times \mathrm{MAP}-0.052\times \text{LVSP}-4.517\times \mathrm{ABF} \end{array}$$

where *P* is the probability of AVC. The model was of statistical significance (χ^2^ = 85.822, *p* < 0.001). The accuracy (consistency) of the model was 88.7%, sensitivity was 82.6%, specificity was 94.1%, positive predictive value was 92.7%, and negative predictive value was 85.7%. The Hosmer‐Lemeshow goodness‐of‐fit test yielded a chi‐square value of 9.834 (*p* = 0.277 > 0.05). The ROC curve of the model is shown in Figure 8.

**TABLE 2:** Dichotomous logistic regression analysis

Variables	*β*	OR	95% CI of OR	*Statistic*	*p*
Constant	−24.04	0.00	0.00–0.00	−2.35	0.019
ABF (L/min)	−4.52	0.01	0.00–0.09	−3.42	0.001
LVSP (mm Hg)	−0.05	0.95	0.91–0.98	−2.97	0.003
MAP (mm Hg)	−0.14	0.87	0.75–0.98	−1.99	0.046
PRS (rpm)	0.02	1.02	1.01–1.04	3.42	0.001
PP (mm Hg)	−0.19	0.82	0.68–0.96	−2.23	0.026

Abbreviations: ABF, artificial vascular blood flow; CI, confidence interval; LVSP, left ventricular systolic pressure; MAP, mean arterial pressure; OR, odd ratios; PP, pulse pressure; PRS, pump rotate speed; *β*, regression coefficients.

## 4 DISCUSSION

**FIGURE 9:**
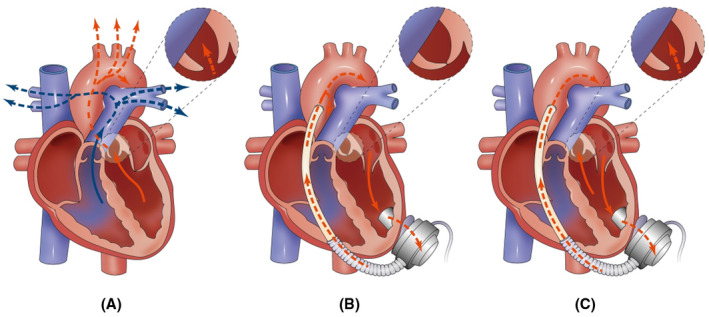
Hemodynamics in the normal heart after CF‐LVAD implantation. (A) Normal heart: The left ventricle contracts, blood enters the aorta through the aortic valve; (B) the aortic valve is completely closed, the natural left ventricle and CF‐LVAD work in series model, and blood enters the aorta via CF‐LVAD; (C) aortic valve opening or intermittent opening, natural left ventricle, and CF‐LVAD work in “parallel”, part of the blood enters the aorta through the aortic valve)

According to the latest report of INTERMACS, CF‐LVAD is preferred during clinical practice as an alternative to pulsatile flow pump.^1^, ^2^ It has been shown that under non‐pulsatile circulation, around 86.2% of patients receiving CF‐LVAD develop persistent AVC.^12^ As displayed in Figure 9, during AVC, the left ventricle and CF‐LVAD can work in series model (Figure 9B); a significant change in the aortic valve hemodynamics was observed secondary to the pulsatile flow generated from natural ventricular contraction and aortic root pressure.^3^, ^13^ It has been established that AVC is associated with arterial pulsatility weakening, vascular wall remodeling, increased inflammatory reaction, gastrointestinal bleeding, and aortic insufficiency (AI).^14^, ^15^, ^16^, ^17^ Dobarro et al. reported that AVC could cause a decrease in the overall survival rate in patients supported with CF‐LVAD, inducing damage to the left ventricle.^18^ With CF‐LVAD support, partial ventricular unloading is more conducive to the recovery of left ventricular function than full support unloading.^19^

In the present study, a CF‐LVAD model was established in Small Tail Han sheep, and the experimental results were interpreted from observational and statistical perspectives. During the observational study, we found that under a constant HR, the ABF increased with increased pump speed while the MAP marginally changed. Besides, the pump speed affected aortic valve closure. During the statistical analysis, the increase in pump speed was indicative of a higher risk of AVC (OR = 1.02, 95% CI = 1.01–1.04, *p* = 0.001), consistent with the literature.^20^, ^21^, ^22^, ^23^ Under normal circumstances, the aortic valve closes when the LVP falls below the AOP. The PD is considered a direct determinant of the aortic valve functional status. However, we found that after CF‐LVAD implantation, the risk of AVO was still present with a negative PD (Figures 4 and 5). Statistical analysis showed that using PD alone to predict AVC can lead to a missed diagnosis. We hypothesize that PD alone is insufficient for the prediction of AVC and that changes in PD after CF‐LVAD implantation may be related to the pump speed, HR, and/or ABF.

Moreover, no consensus has been reached on the relationship of HR with pump flow and aortic valve functional status. Akimoto et al. reported that at a constant pump speed, the average pump flow was positively correlated to the HR.^24^ In contrast, Muthiah et al. revealed CF‐LVAD pump flow was independent of the HR.^25^ In the present study, we found that the pump flow transiently increased and then decreased following an increase in HR at a constant pump speed. Besides, the increase in HR also affected the functional status of the aortic valve. It is widely acknowledged that HR is related to the opening and closing of the aortic valve due to inertia of valve leaflet movement. After CF‐LVAD implantation, an increased HR at a constant pump speed resulted in decreased cardiac stroke output, blood flow from the pulmonary vein to the left ventricle, LVSP and ABF, leading to AVC. Therefore, it is believed that the indirect effect of HR on the functional status of the aortic valve is more significant after CF‐LVAD implantation. In the present study, the HR exhibited no significant value in predicting the functional status of the aortic valve during multivariate analysis. Considering the observed trends in ABF and pressure (Figure 6), we hypothesize that HR may indirectly affect blood pump flow and LVSP by affecting the preload or afterload of the blood pump, which in turn causes AVC.

Moreover, Imamura et al. revealed that the contractibility of the left ventricle was associated with aortic valve opening in patients with CF‐LVAD support.^26^ In our study, LVSP was negatively associated with the risk of AVC (OR = 0.95, 95% CI = 0.91–0.98, *p* = 0.003), suggesting that a lower LVSP indicated a higher risk of AVC. Under normal circumstances, the aortic valve opens in response to increased left ventricular pressure to overcome aortic pressure.^27^ Following implantation of CF‐LVAD, the increased left ventricular unloading induced by the higher pump speed leads to larger pressure differences between the aorta and the left ventricle, resulting in AVC.^28^

In a simulation of in vitro circulation by Tuzun et al., increased pump speed was paralleled by an increased pump flow and higher MAP, which led to AVC.^4^, ^9^ In our study, AVC was negatively associated with the ABF and MAP (OR < 1). It is highly conceivable that other factors affect the functional status of the aortic valve, ABF, and MAP. From the flow curve, it can be observed that the ABF was affected by the pump speed and HR. Additionally, MAP was stable when the pump speed or ABF increased over a certain range, consistent with a study by Hayward et al.,^29^ which expounded a linear relationship between the pump speed and flow; the flow was stable over a relatively small range. The above findings suggest that, in vivo, the compliance and feedback mechanism of the arterial wall could help avoid MAP fluctuations caused by pump speed changes to some extent.

It has been established that PP can indicate the amplitude of pressure fluctuations in a cardiac cycle, and decreases in PP usually result from AVC. Myers et al.^30^ studied 35 cases supported with Jarvik 2000 LVAD and reported PP values greater than 15 mm Hg, which were within the safety range in most patients. In a prospective study by Kohno et al. that enrolled 85 cases with Jarvik 2000 LVAD, the multivariate analysis showed an association between postoperative AI and PP (HR = 1.06, 95% CI = 1.00–1.13, *p* = 0.045).^31^ Consistently, we found that a decline in PP was associated with AVC (OR = 0.82, 95% CI = 0.68–0.96, *p* = 0.026).

Using CF‐LVADs such as HeartMate III, HeartWare and Jarvik 2000, periodic reduction of pump speed (intermittent low‐speed mode) can be applied to promote the intermittent opening of the aortic valve. However, whether this mode is conducive to AVO or preventive to AI development remains to be studied in larger cohorts.^31^ Saeed et al. reported that using the intermittent low‐speed mode, only 65% of patients developed persistent AVO.^32^ Currently, HeartCon is on the verge of clinical application. The present study sought to establish a predictive model for AVC after CF‐LVAD implantation using experimental animal data, hoping to provide novel insights for further out‐of‐hospital self‐assessment of the functional status of the aortic valve. The ultimate goal is individualized surveillance of patients and pump speed optimization. Our model yielded good discrimination and calibration performances with an AUC of 0.973 (95% CI = 0.978–0.995 and a *P*‐value >0.05 in the Hosmer‐Lemeshow goodness‐of‐fit test). When HeartCon is applied on a larger scale, data from multi‐center clinical studies can be used to validate and improve our model.

**FIGURE 10:**
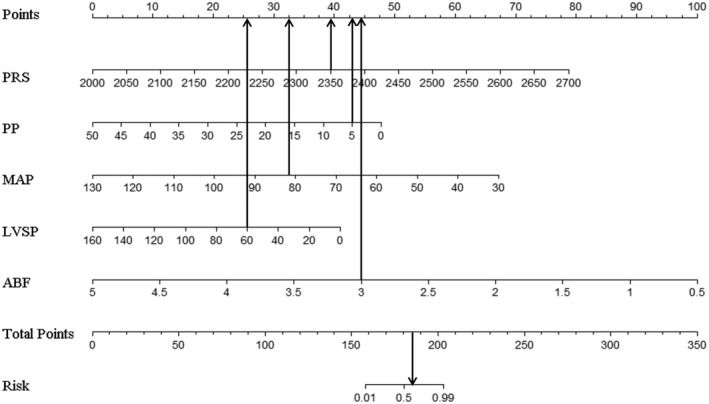
The example of nomogram. ABF, artificial vascular blood flow; LVSP, left ventricular systolic pressure; MAP, mean arterial pressure; PP, pulse pressure; PRS, pump rotate speed

Changing the pump speed or other parameters is generally not recommended during clinical practice if no close surveillance is available. However, with the establishment of our model, discharged patients could be assessed to know the functional status of the aortic valve, which could optimize the scheduling of follow‐ups and re‐examinations. Guided by transthoracic echocardiography, individualized adjustment based on hemodynamics parameters for CF‐LVAD parameters can be obtained to promote AVO and decrease the incidence or progression of relative complications.^33^, ^34^ For instance, for a discharged patient with the following parameters pump speed 2350 rpm, ABF 3 L/min, LVSP 60 mm Hg, PP 5 mm Hg, and MAP 82 mm Hg, the corresponding score for each variable would be 40, 45, 25, 42.5, and 32.5, resulting in a total score of 185 and a risk for AVC of more than 0.5 (Figure 10), implying that the patient has a probability of at least 50% to sustain AVC. In this case, the patient should schedule an appointment to adjust pump parameters and maintain AVO.

In vivo, hemodynamics are well recognized to be affected by multiple factors, including pump speed, preload, left ventricular and aortic compliance, and peripheral vascular resistance. The relationships among pump speed, HR, and ABF are unknown, nor is it clear whether ABF can influence MAP. PD is reportedly directly caused by AVC. However, prediction of aortic valve functional status by PD alone increases susceptibility to missed diagnoses. Statistical analysis showed that the sensitivity of PD alone in predicting the aortic valve functional status was lower than our nomogram (65.2% vs. 82.6%). Importantly, we found that it is feasible to predict aortic valve functional status using a combination of pump speed, LVSP, ABF, MAP, and PP.

Inevitably, several limitations were found in this study. First, a total of 97 observational data were from 5 sheep, and the relevance between adjacent data cannot be completely ruled out, although great efforts were made in the design of our experiment, and the independence of each observed variable was statistically proven. Further experiments with larger sample sizes conducted in multiple centers should be devised. Nonetheless, our model for predicting the risk of AVC has a certain application value. Besides, the long‐term effect of CF‐LVAD on the functional status of the aortic valve is unknown, warranting further experiments in animal models of chronic heart failure. Moreover, CF‐LVAD is usually applied clinically by an anastomosis between the outflow tract of the device and the ascending aorta of the recipient. In contrast, in the present study, the descending aorta underwent anastomosis since the ascending aorta of Small Tail Han sheep is relatively short. This finding could have led to minor hemodynamic differences.

## 5 CONCLUSION

After CF‐LVAD implantation, pump speed, HR, LVP, ABF, and MAP can directly or indirectly affect PD, affecting the functional status of the aortic valve. Among these, pump speed, LVSP, ABF, MAP, and PP are significant predictors for the risk of AVC. Predictive models built from these predictors yielded good performance in distinguishing between AVO and AVC after CF‐LVAD implantation. Future studies should emphasize developing new approaches for effective out‐of‐hospital prediction of aortic valve functional status for individualized hemodynamic optimization, which may be more beneficial to improving patient outcomes than only monitoring pump speed.

## CONFLICT OF INTEREST

All authors had no conflicts of interest to disclose.

## AUTHOR CONTRIBUTIONS

Xin‐Yi Yu: Design, Data collection, Data analysis, Data interpretation, Drafting article, and Critical revision of article. Jian‐Wei Shi and Yi‐Rui Zang: Data collection, Data interpretation, and Drafting article. Jie‐Min Zhang: Concept/design, Data analysis, Data interpretation, and Critical revision of article. Zhi‐Gang Liu: Concept/design, Data analysis, Data interpretation, Critical revision of article, and Approval of article.
